# Pathogenesis of Molar Hypomineralisation: Aged Albumin Demarcates Chalky Regions of Hypomineralised Enamel

**DOI:** 10.3389/fphys.2020.579015

**Published:** 2020-09-30

**Authors:** Vidal A. Perez, Jonathan E. Mangum, Michael J. Hubbard

**Affiliations:** ^1^Department of Pharmacology and Therapeutics, The University of Melbourne, Parkville, VIC, Australia; ^2^Department of Pediatric Stomatology, Faculty of Health Sciences, University of Talca, Talca, Chile; ^3^Department of Paediatrics, The University of Melbourne, Parkville, VIC, Australia; ^4^Faculty of Medicine Dentistry and Health Sciences, The University of Melbourne, Parkville, VIC, Australia; ^5^Melbourne Dental School, The University of Melbourne, Parkville, VIC, Australia

**Keywords:** global health, paediatric disorders, dental defects, dental caries, medical prevention, developmental biomarkers, serum albumin, biomineralisation

## Abstract

Molar hypomineralisation (MH) is becoming globally recognised as a significant public health problem linked to childhood tooth decay. However, with causation and pathogenesis unclear after 100 years of investigation, better pathological understanding is needed if MH is to become preventable. Our studies have implicated serum albumin in an extracellular pathomechanism for chalky enamel, opposing longheld dogma about systemic injury to enamel-forming cells. Hypothesising that chalky enamel arises through developmental exposure to serum albumin, this study used biochemical approaches to characterise demarcated opacities from 6-year molars. Addressing contradictory literature, normal enamel was found to completely lack albumin subject to removal of surface contamination. Querying surface permeability, intact opacities were found to lack salivary amylase, indicating that “enamel albumin” had become entrapped before tooth eruption. Thirdly, comparative profiling of chalky and hard-white enamel supported a dose-response relationship between albumin and clinical hardness of opacities. Moreover, albumin abundance delineated chalky enamel from white transitional enamel at opacity borders. Finally, addressing the corollary that enamel albumin had been entrapped for several years, clear signs of molecular ageing (oxidative aggregation and fragmentation) were identified. By establishing aged albumin as a biomarker for chalky enamel, these findings hold methodological, clinical, and aetiological significance. Foremost, direct inhibition of enamel-crystal growth by albumin (here termed “mineralisation poisoning”) at last provides a cogent explanation for the clinical presentation of demarcated opacities. Together, these findings justify pursuit of an extracellular paradigm for the pathogenesis of MH and offer exciting new prospects for alleviating childhood tooth decay through medical prevention of MH.

## Introduction

Popularly known as “chalky teeth,” molar hypomineralisation (MH) is becoming globally recognised as a significant public health problem linked to childhood tooth decay (Hubbard et al., 2017; Hubbard, 2018).^1^^,^^2^ Defined by discoloured spots or patches of porous enamel (“demarcated opacities”) on one or more molars, MH puts sufferers at risk of toothache and unusually rapid decay. In moderate and severe cases where the opacities have a chalky texture (Gottlieb, 1920; Hurme, 1949; Myers, 1955), the enamel surface often fails soon after tooth eruption, providing a hygiene-resistant nidus for dental plaque. Accelerated decay (comprising acid attack from dental caries and diet, plus disintegration under chewing forces) may then invade the porous chalky enamel, frequently leading to costly management needs (e.g., ongoing restorations, tooth extraction, and orthodontics). Clinically, it would be useful to know more about chalky opacities, particularly regarding their prognosis and delineation from hard-white opacities which are tougher and less prone to decay. Preventively, it appears that much childhood decay is at stake because MH affects the 2-year molars and/or 6-year molars of 1-in-5 children worldwide.^3^ With the likelihood that MH is developmentally acquired and so potentially preventable, better aetiological understanding is paramount (Suckling, 1989; Mangum et al., 2010a; Hubbard et al., 2017; Hubbard, 2018).

Although causation and pathogenesis of MH remain unclear after 100 years of investigation into chalky enamel, recent biochemical investigations have opened an enticing new direction for aetiological research (Gottlieb, 1920; Suckling et al., 1976; Mangum et al., 2010a; Perez et al., 2018; Williams et al., 2020). Demarcated opacities have long been thought to arise from systemic injuries to enamel-forming cells (ameloblasts) during the hardening (maturation) stage of enamel formation (Suckling, 1989; Suga, 1989; Jalevik et al., 2001; Weerheijm, 2003; Alaluusua, 2010; Babajko et al., 2017). However, this proposition has failed to provide mechanistic explanations for several fundamental characteristics of chalky opacities (e.g., chalkiness, topography, and sporadic presentation). An alternative pathomechanism involving localised exposure of immature enamel to serum albumin was suggested following proteomic comparison of opacities bearing intact and broken surfaces (Mangum et al., 2010a). While attractive, such an extracellular mechanism faced longstanding concerns about the propensity for albumin to associate with porous enamel artefactually (Couwenhoven et al., 1989; Strawich and Glimcher, 1989; Chen et al., 1995; Wright et al., 1997; Takagi et al., 1998; Farah et al., 2010; Robinson, 2014). In a partner study querying medical onset of MH, we addressed this issue using a novel “molecular timestamping” approach and obtained strong evidence that albumin was incorporated in chalky enamel developmentally (Williams et al., 2020). With artefact concerns allayed, it became appropriate to further evaluate albumin infiltration as a central mechanistic element of chalky enamel development from a clinical perspective.

Aiming to complement the medical-onset findings, this study adopted a dental-outcome perspective and characterised albumin residing within chalky opacities. Seeking evidence for a mechanistic role in enamel hypomineralisation and hypothesising direct inhibition of enamel-crystal growth (Robinson et al., 1992, 1994; Mangum et al., 2010a), we analysed correlations between albumin abundance and clinical hardness of enamel. Using 6-year (first permanent/adult) molars, comparative protein-profiling was done on chalky opacities, hard-white opacities, and normal enamel. Ageing-related molecular alterations of albumin, and permeability of the opacity surface, were also investigated, addressing the corollary that albumin had been entrapped for several years before tooth eruption.

## Materials and Methods

### Specimens, Biologicals, and Biochemicals

Extracted 6-year molars were collected with informed consent under institutional ethical approval (HEC 0719683, University of Melbourne). Straight after extraction the teeth were rinsed in physiological saline and patted dry with gauze, then stored unfixed at −80°C as before (Mangum et al., 2010a; Williams et al., 2020). For oral fluid analysis, stimulated saliva was collected from healthy adults chewing on paraffin wax, then clarified by centrifugation (22,000 *g* for 5 min at 4°C) before proteins were precipitated with ethanol and dissolved in sodium dodecyl sulfate-polyacrylamide gel electrophoresis (SDS-PAGE) sample buffer. Human albumin (Sigma), antibodies against human albumin (rabbit polyclonal, product PAB10220 from Abnova), and human alpha-amylase 1 (mouse polyclonal, product ab171807, from Abcam), were sourced commercially. All other biochemicals and reagents were of analytical grade.

### Preparation of Enamel Samples

A paediatric dentist (VP) diagnosed MH using standard criteria for demarcated opacities (Suckling, 1998; Weerheijm et al., 2003). Opacities bearing a visibly intact (shiny) surface were selected for analysis and those with surface breakdown (cracking, chipping, pitting, or caries involvement) were excluded to avoid contamination by oral fluid proteins (Mangum et al., 2010a). To remove surface-associated protein, teeth were cleaned by washing in phosphate solution (0.4 M NaH_2_PO_4_ pH 7.2) for 3 min, then surface-abraded at 5,000 rpm with a dental polishing disk (Sof-Lex 1982C, from 3M Australia) until perikymata were no longer observable under 20x magnification. After surface-cleaning, hard enamel (visibly normal and hard-white) was harvested with a slowly rotating dental bur (No. 2 tungsten carbide, from Komet), and chalky enamel was removed with a hand excavator. Ensuing samples of powdered enamel were quantified volumetrically with a calibrated 1-μl micro-spoon (Fine Science Tools) before immediate preparation for SDS-PAGE.

### Profiling of Enamel Proteins

Proteins were isolated from 14 opacities (representing 11 teeth from 11 cases, no overlap with partner study; Williams et al., 2020) and normal-enamel controls using acid precipitation and solubilisation at room temperature in reducing SDS-PAGE sample buffer as before (Mangum et al., 2010a). Briefly, enamel powder (typically 2–5 μl) was extracted with 10 vols 10% trifluoroacetic acid and the resulting pellet solubilised in 10 vols SDS-PAGE sample buffer containing protease inhibitors (1 mM dithiothreitol, 1 mM benzamidine, 1 mM phenylmethylsulfonyl fluoride, 5 μg/ml pepstatin, and 5 μg/ml leupeptin). Only freshly prepared samples were used to avoid artefactual losses reported earlier (Mangum et al., 2010a). Equivalent enamel volumes were analysed by SDS-PAGE using precast mini-gels (AnyKDa mini-protean TGX, from BioRad, with Tris/glycine buffer) followed by Coomassie Blue staining. Protein size (*M*_r_ expressed as kDa for brevity) was calibrated with a prestained ladder (Precision Plus Dual Colour Protein standards, from BioRad), and average nominal values for serum albumin (55 kDa) and enamel albumin (65 kDa) were derived by semi-log plot. Note these values differ from classical determinations made with unstained protein ladders (Mangum et al., 2010a). For immunoblotting, proteins were electrotransferred to 0.2 μm nitrocellulose membrane using a Trans-Blot Turbo system (BioRad) and immunospecific bands were detected colorimetrically (Vectastain ABC alkaline phosphatase and peroxidase kits, from Vector Labs; Mangum et al., 2010a; Perez et al., 2018). Standard antibody dilutions were: anti-albumin 1:2,000 and anti-amylase 1:1,000. Effects of dithiothreitol (DTT; from Sigma) and tris(2-carboxyethyl) phosphine (TCEP; from Pierce) were assessed by solubilising enamel proteins in SDS-PAGE sample buffer that lacked both of these reducing agents, then subsequently had reducers added alone or in combination as indicated in the figure legends. Protein bands were quantified using semi-quantitative imaging densitometry as before (Perez et al., 2018).

### Other Methods

Mineral-binding activity was assayed by incubating proteins with powdered pure hydroxyapatite before centrifuging to separate bound and unbound fractions, as previously (Mangum et al., 2010a; Perez et al., 2018). Proteins were identified from Coomassie-stained gel bands by peptide mass fingerprinting as before (Mangum et al., 2010a) except that a different mass spectrometer (Orbitrap elite ETD Thermo Scientific) was used. Digital image manipulation was limited to linear brightness and contrast adjustments at whole gel/blot level, and selected areas were composited as described in the figure legends. Original images of whole gels/blots were provided for review.

## Results

### Albumin Is Absent From Normal Bulk Enamel

**Figure 1:**
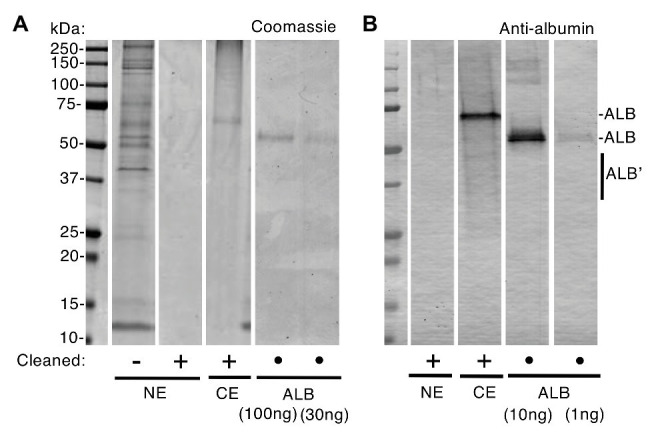
Albumin is absent from normal enamel after surface-cleaning. Clinically normal enamel (NE) was harvested from a 6-year molar before and after surface-cleaning as indicated (• = not applicable), then subjected to sodium dodecyl sulfate-polyacrylamide gel electrophoresis (SDS-PAGE) and albumin-immunoblotting with peroxidase detection as described under “Materials and Methods” section. Chalky enamel (CE) from an intact opacity on the same tooth, and fresh serum albumin (ALB), were processed in parallel. Equal amounts of NE (equivalent to CE) were loaded in **(A)** and **(B)**. **(A)** Coomassie Blue staining revealed numerous protein bands in normal enamel that had not been surface-cleaned, yet no proteins were detectable after surface-cleaning. In contrast, chalky enamel contained a single major band after surface-cleaning (identified as albumin, in **B**). The ALB standards verified linearity of detection with a sensitivity of about 30 ng. **(B)** Immunoblotting verified an absence of albumin in cleaned normal enamel, at 1 ng sensitivity (i.e., 30-fold higher than in **A**). Note that, in addition to the major albumin bands, fragmented albumin species (ALB’) were visible in chalky enamel. This figure was composited from **(A)** two Coomassie-stained gels, and **(B)** an immunoblot, made in parallel from one gel in **(A)**.

**Figure 2:**
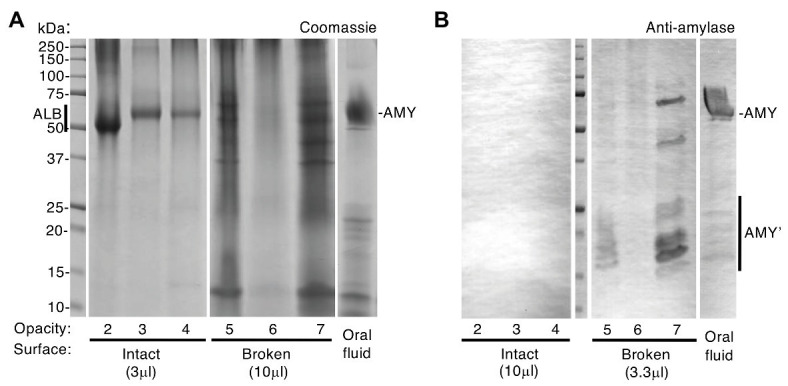
Intact opacities are impermeable to amylase, unlike broken opacities. Chalky enamel harvested from three opacities with intact surfaces and three with visibly broken surfaces (opacities 2–4 and 5–7, respectively) was subjected to SDS-PAGE and amylase immunoblotting with phosphatase detection as indicated. Differential sample loads were used for semi-quantification as indicated, and oral fluid was analysed in parallel. **(A)** Coomassie Blue staining revealed a single major band [albumin (ALB)] in intact opacities that had been surface-cleaned, consistent with Figure 1. In contrast, broken opacities exhibited a complex banding pattern overlapping that of oral fluid, in which amylase (AMY) predominated as expected. **(B)** Immunoblotting revealed amylase (AMY) in broken opacities but not in intact opacities, despite 3-fold up-loading of the latter as indicated. Amylase fragments (AMY’) were more prominent in broken enamel than in oral fluid. Equal amounts of oral fluid (10 μl equivalent) were loaded in **(A)** and **(B)**. This figure was composited from **(A)** two Coomassie-stained gels, and **(B)** a single immunoblot made in parallel.

**Figure 3:**
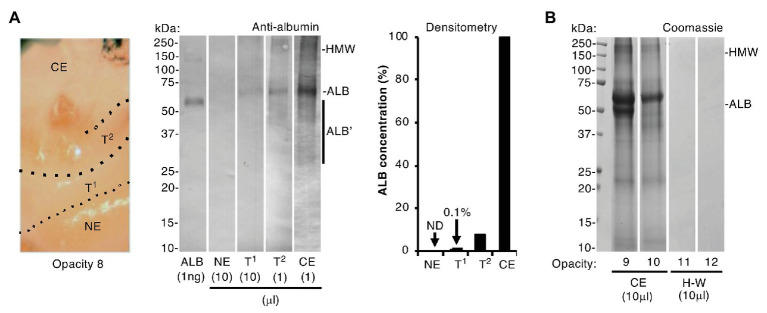
Albumin differentiates chalky enamel from hard-white enamel. Intact chalky and hard-white opacities (opacities 8–10 and 11–12, respectively) were subjected to SDS-PAGE and albumin-immunoblotting (phosphatase detection, with <1 ng sensitivity) as indicated. **(A)** (*Left panel*) Photograph showing how opacity 8 was subdissected to provide samples of chalky enamel (CE), visibly normal enamel (NE), and intervening hard-white enamel referred to here as transitional zones 1 and 2 (T1, T2; Mahoney et al., 2004). (*Centre panel*) Immunoblot analysis, employing differential loads of opacity 8 samples as indicated, showed that albumin was much less prevalent in the transitional hard-white regions nearing the opacity border than in chalky enamel. Albumin fragments (ALB’) and high molecular weight aggregates (HMW) were visible as indicated. (*Right panel*) Densitometry of the immunoblot showed striking (10–100-fold) differences in albumin content between chalky and transitional enamel as indicated [none detected (ND)]. **(B)** Coomassie Blue staining showed that hard-white (H-W) opacities 11 and 12 were devoid of albumin, in striking contrast to chalky opacities 9 and 10 (CE), which again exhibited albumin fragmentation and aggregation. This figure was composited from **(A)** two immunoblots and **(B)** two Coomassie-stained gels.

**Figure 4:**
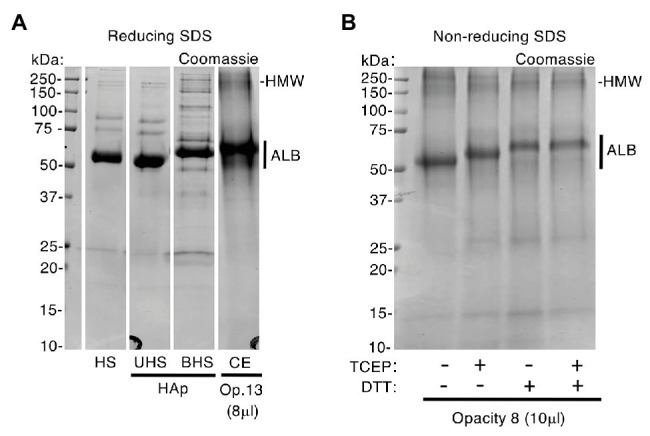
Enamel albumin differs from fresh serum albumin. An excess of human serum (HS) was exposed to hydroxyapatite (HAp) yielding unbound and bound fractions (UHS, BHS) as described under Methods. Samples of serum, chalky enamel (CE), and fresh serum albumin were then subjected to SDS-PAGE under reducing (100 mM DTT and/or 4 mM TCEP in SDS loading buffer) and non-reducing conditions (no DTT or TCEP), followed by Coomassie Blue staining as indicated. **(A)** After being bound to hydroxyapatite, albumin from human serum (BHS) exhibited reduced mobility, matching that of enamel albumin (CE; opacity 13). In contrast, the unbound albumin showed unchanged mobility when compared with starting serum (UHS and HS, respectively) and fresh albumin standard (not shown). **(B)** In absence of DTT and/or TCEP (*leftmost lane*), enamel albumin from opacity 8 migrated faster (compare with **A**), matching the fresh albumin standard (not shown). Reduction with TCEP induced an intermediate mobility state when compared with DTT. The high molecular weight albumin bands (HMW; see also Figure 3) were enhanced in the absence of reducers, consistent with disulfide-based aggregation. Sample loads were 8 and 10 μl (i.e., 0.8 and 1 μl enamel-powder equivalents) for opacity 13 and 8, respectively. This figure was composited from a **(A)** a single Coomassie-stained gel, and **(B)** a second Coomassie-stained gel.

Differences between normal and diseased tissues are fundamental to diagnosis and aetiology yet the question of whether albumin is a normal constituent of enamel has been contentious for many years (Couwenhoven et al., 1989; Strawich and Glimcher, 1989; Robinson et al., 1992, 1994; Chen et al., 1995; Wright et al., 1997; Takagi et al., 1998; Robinson, 2014). In 2010, a proteomic study reignited this question having found significant amounts of albumin in clinically normal enamel (Farah et al., 2010). Conversely, our proteomic data indicated albumin was effectively absent (Mangum et al., 2010a), leading us to ask whether varying amounts of surface contamination could underlie these conflicting observations. An initial experiment showed that albumin was rapidly adsorbed by hydroxyapatite (t_0.5_ < 45 s; Supplementary Figure S1), implying that only brief exposures would be required for contamination of surface enamel by blood or saliva (oral fluid). Accordingly, before harvesting enamel specimens, we applied a surface-cleaning procedure comprising a phosphate wash and mild abrasion to remove perikymata (surface growth lines). When superficial normal enamel was viewed by SDS-PAGE with Coomassie staining, a variety of protein bands were obvious in uncleaned teeth but absent after surface-cleaning (Figure 1A). Immunoblotting with 30-fold-higher sensitivity showed a lack of albumin in surface-cleaned normal enamel, whereas albumin predominated in chalky enamel (Figure 1B) as expected (Mangum et al., 2010a). By comparison with 12 other opacities (Figures 2–4), it was established that the (undetectable) amounts of albumin in normal enamel were at least 100-fold lower than those in chalky enamel. We concluded that, in context of MH, albumin is effectively absent from normal bulk enamel.

### Albumin Is Entrapped by Impermeable Surface Enamel Before Tooth Eruption

Now able to regard albumin-containing enamel as abnormal, attention turned to the origin of “enamel albumin” – being that present in chalky opacities with intact surfaces (Mangum et al., 2010a). As illustrated elsewhere (Figure 1A; Williams et al., 2020), we questioned whether serum albumin had either (1) been incorporated developmentally then entrapped under an impermeable layer of surface enamel or (2) had infiltrated pre-existing porous enamel through visibly-intact yet protein-permeable surface enamel. Although the first option had been proven true by our investigation of alpha-fetoprotein – the foetal isoform of serum albumin (Williams et al., 2020) – the possibility remained that a lesser proportion of adult serum albumin was acquired later during tooth eruption or extraction. Our earlier comparison of intact and broken opacities argued against the second option because oral fluid proteins appeared unable to infiltrate intact opacities (Mangum et al., 2010a). Here, the latter analysis was repeated with a different set of six specimens (Figure 2A) and again Coomassie staining showed that broken opacities contained numerous proteins, unlike intact opacities where albumin was uniquely predominant. Probing deeper, we used immunoblotting to profile amylase, an abundant salivary protein that binds hydroxyapatite avidly like albumin (Johnsson et al., 1993). As shown in Figure 2B, amylase was detected in broken opacities but not in intact opacities despite a 3-fold higher loading of the latter. Interestingly, broken opacities preferentially retained fragments of amylase, suggesting their smaller size enabled better penetration of enamel porosity. Using more-sensitive conditions (2.5x higher antibody concentration, intact/broken loading ratio of 33), amylase was again undetectable in intact opacities (not shown). We concluded that visibly-intact opacities are impermeable to protein, which serves both to entrap developmentally acquired albumin and to exclude external proteins from binding to porous subsurface enamel.

### Albumin Is Abundant in Chalky Regions of Hypomineralised Enamel

Collective strength of the foregoing results (Figures 1, 2, plus; Williams et al., 2020) led us to accept that exposure of developing enamel crystals to albumin likely plays a direct inhibitory role in hypomineralisation, as we and others speculated earlier (Robinson et al., 1992, 1994; Farah et al., 2010; Mangum et al., 2010a). Hereafter we term this process “mineralisation poisoning,” noting that a poisoning metaphor accords with crystallographic terminology and also leaves open the possibility of rescue. It followed that, if albumin acts as a poison, then correspondence between amount of enamel albumin and degree of chalkiness would be expected. We evaluated this dose-response relationship firstly by comparing chalky and hard areas within individual opacities, and secondly by comparing whole opacities of varying hardness (chalky vs. hard-white). Sub-sampling of a representative opacity is illustrated in the *left panel* of Figure 3A, whereby chalky enamel was harvested from the centre by hand tool, and two hard-white zones bordering normal enamel (“transitional enamel”; Mahoney et al., 2004) were removed with a dental bur. As revealed by immunoblotting (Figure 3A, *centre panel*), albumin abundance declined progressively from chalky enamel through the transitional zones to become undetectable in normal enamel. Densitometry showed a sharp (≈10-fold) decline passing from chalky enamel to the first (densely opaque) transition zone, and this decreased >50-fold further in the second transition zone where opacity faded (Figure 3A, *right panel*). When whole opacities were profiled with Coomassie staining (Figure 3B), hard-white opacities were devoid of any protein in striking contrast to the albumin-enriched chalky opacities. We concluded that albumin is abundant in chalky enamel but not in hard-white enamel, grossly supporting a dose-response relationship with poisoned mineralisation.

### Entrapped Albumin Undergoes Molecular Ageing

The above findings implied that several years would have passed between albumin entrapment during infancy (i.e., onset of MH) and subsequent harvesting of opacities from extracted 6-year molars. If so, does enamel albumin show signs of such history? Albumin is known to undergo molecular changes as it ages, including fragmentation, oxidation and aggregation (Donadio et al., 2012; Sancataldo et al., 2014). It was evident from SDS-PAGE analysis that enamel albumin migrated more slowly than fresh serum albumin under standard (reducing) conditions (Figures 1, 3, 4). Asking whether this anomaly was due to molecular ageing or the extra steps taken for isolation from enamel, blood serum was exposed to hydroxyapatite then acid-solubilised as for enamel albumin (Figure 4A). Strikingly, in the hydroxyapatite-bound fraction, serum albumin had lower mobility like enamel albumin, whereas its mobility was normal in the unbound fraction. The distinctive mobility of enamel albumin was therefore ascribed to a procedural effect, not ageing. When the experiment was repeated in non-reducing conditions, enamel albumin exhibited the same mobility as fresh albumin (Figure 4B; *first lane*) prompting consideration of oxidative effects. Knowing that oxidised albumin can form oligomers (Sancataldo et al., 2014), the detection of high molecular weight (HMW) bands in most opacities seemed indicative (Figures 2–4). We confirmed the presence of albumin in HMW bands both by immunoblotting (Figure 3) and mass spectrometry (Supplementary Figure S2). Moreover, the amount of HMW-albumin decreased substantially when SDS-PAGE samples were solubilised with two types of reducing agents (dithiothreitol and TCEP), alone and in combination (Figure 4B). These results indicate that chalky enamel contains oxidatively-crosslinked aggregates of albumin, consistent with prolonged ageing. A third obvious difference was the distinctive fragmentation of enamel albumin (Figures 1B, 3A), as characterised previously by mass spectrometry (Mangum et al., 2010a). We concluded from the fragmentation and aggregation data that enamel albumin was markedly aged, consistent with having been trapped in chalky enamel for years.

## Discussion

Improved pathological understanding of MH is needed if its worldwide impact on childhood tooth decay is to be alleviated. This dentally focussed study correlated serum albumin with chalky regions of demarcated opacities by showing that hard-white enamel has vastly lower amounts of albumin, and that normal enamel has none if surface contamination is excluded. In intact opacities, enamel albumin was found to be molecularly aged and isolated from the oral environment, consistent with having been fully acquired and entrapped during tooth development. Together, the findings from this and a partner study addressing medical onset (Williams et al., 2020) justify pursuit of an extracellular “mineralisation poisoning” paradigm for the pathogenesis of MH. In essence, we propose that serum albumin: (1) infiltrates immature enamel and survives the proteolytic conditions therein, unlike the principal enamel protein, amelogenin; then (2) binds to enamel crystals and stalls their growth, collectively leading to porous chalky enamel. This pathomechanism and recognition of aged albumin as a biomarker for chalky enamel holds clinical, aetiological and methodological significance, as follows.

Methodologically, our findings reinforce the need for careful segregation of intact opacities from those with broken surfaces, and also to be watchful for contamination when using high-sensitivity analyses, such as immunoblotting and mass spectrometry (Mangum et al., 2010a,b). The evidence that significant amounts of surface-adsorbed proteins can accrue rapidly and be carried over to samples of bulk enamel (Figure 1; Supplementary Figure S1), together with multiple reports that the natural enamel pellicle contains numerous proteins including albumin (e.g., Ventura et al., 2017), suggests that surface-cleaning procedures should be adopted routinely. It seems plausible that such contamination contributed to proteomic findings of albumin in normal enamel (Farah et al., 2010; Castiblanco et al., 2015; Jagr et al., 2019), particularly if superficial enamel had been enriched through shallow sampling. Moreover, by confirming the governing influence of intact surface enamel (Mangum et al., 2010a), the amylase results (Figure 2) highlight the need for clinicians and scientists to collaborate on diagnostic guidelines for future translational research (Hubbard et al., 2017; Kirkham et al., 2017).^4^

Aetiologically, the new findings about enamel albumin reinforce our previous biochemical findings (Mangum et al., 2010a; Williams et al., 2020) and so justify further attention to the role mineralisation poisoning plays in MH pathogenesis. Together, the findings that enamel albumin shows two distinct signs of ageing (fragmentation and oxidative aggregation; Figure 4), and that post-eruptive absorption from oral fluid or blood can be discounted in intact opacities (Figure 2), accords with albumin having been acquired during enamel development and not later. A third sign of molecular ageing, namely a high proportion of oxidised methionine residues (Sancataldo et al., 2014), was found during the mass spectrometry analysis (Supplementary Figure S2 and data not shown) and warrants further attention. Comparison of chalky and hard-white enamel (Figure 3) suggests that a threshold amount of albumin must accumulate for enamel to become chalky, consistent with a dose-response effect for the poisoning of mineralisation. With albumin identified as the predominant mineralisation inhibitor in chalky enamel, new questions arise regarding the diverse clinical presentations of demarcated opacities (e.g., discolouration, size, shape, and biophysical properties). Further biochemical characterisation should refine our current operational definition of chalkiness (i.e., excavatable with hand tools) and so guide further mechanistic enquiry. The discovery of albumin aggregates that persisted under strongly-reducing conditions (Figure 4B) is also noteworthy. It seems plausible that enamel albumin became insoluble as a result of amorphous aggregation and/or fibrillation (Sancataldo et al., 2014) or *via* transglutaminase-mediated cross-linking as may occur with serum albumin during blood clotting (Thung et al., 1989). This in turn raises two pressing questions now under investigation – where does enamel albumin originate from (e.g., tissue fluid or haemorrhage), and is albumin alone responsible for poisoning enamel mineralisation?

Clinically with MH, a frequent dilemma involves uncertainty whether intact opacities will remain intact under functional duress (e.g., chewing and cleaning) or break down and become a nidus for dental plaque and decay.^5^ Our finding that amylase fragments were retained in broken opacities, but absent from those with visibly intact surfaces (Figure 2B), raises potential to diagnose early stages of breakdown otherwise invisible to the eye. Combined with knowledge that albumin serves both as a gauge of chalkiness and as a biomarker for the opaque border (Figure 3), this avenue might be pursued to develop prognostic and treatment guidelines for demarcated opacities.

Being reliant on scarce specimens and microscale biochemistry, this study had several inherent limitations, including constraints on experimental duplication (precluding statistical analysis) and enamel-sampling resolution. The focus on albumin in 6-year molars also left questions about other decay-prone teeth (2-year and 12-year molars) and blood-derived proteins. Crucially however, our biochemical analyses have provided striking first-time evidence that albumin in chalky enamel differs from fresh serum albumin, consistent with years of entrapment.

In conclusion, this study complements our ground-breaking study of foetal serum albumin (Williams et al., 2020) by showing that chalky opacities are predominated by molecularly aged (i.e., “old”) albumin entrapped below an impermeable enamel surface. Our results establish a dose-response relationship between albumin and enamel chalkiness and settle longstanding contradictions about the abundance of albumin in normal enamel. These advances hold significance for future clinical management of MH and provide another layer of support for the new pathomechanism we have termed “mineralisation poisoning.” Consequently, after 100 years of enigma, cogent molecular hypotheses can now be envisaged for the clinical phenotypes of demarcated opacities and MH. The ensuing aetiological redirection for chalky enamel – switching from primary focus on injured ameloblasts to enamel matrix infiltrated by serum albumin and potentially other blood-derived proteins – offers exciting new prospects for alleviating childhood tooth decay through medical prevention of MH.

## Data Availability Statement

All datasets presented in this study are included in the article/Supplementary Material.

## Ethics Statement

The studies involving human participants were reviewed and approved by Human ethics committee, University of Melbourne. Written informed consent to participate in this study was provided by the participants’ legal guardian/next of kin.

## Author Contributions

MH and JM contributed to the project conception and design. VP, JM, and MH contributed to the experimental design, labwork, data analysis/interpretation and thesis chapters. MH, VP, and JM contributed to the final manuscript, and read and approved the final manuscript.

### Conflict of Interest

MH is founder/director of The D3 Group for Developmental Dental Defects (thed3group.org), a charitable network.

The remaining authors declare that the research was conducted in the absence of any commercial or financial relationships that could be construed as a potential conflict of interest.

## Footnotes

^1^www.thed3group.org

^2^www.chalkyteeth.org

^3^www.thed3group.org/prevalence.html

^4^www.thed3group.org/the-basics.html

^5^www.thed3group.org/specialist-4.html

## Abbreviations

MH: Molar hypomineralisation
DTT: Dithiothreitol
TCEP: Tris(2-carboxyethyl)phosphine
CE: Chalky enamel
ALB: Serum albumin
NE: Normal enamel
OF: Oral fluid
HMW: High molecular weight
HS: Human serum
SDS-PAGE: Sodium dodecyl sulfate-polyacrylamide gel electrophoresis.
