# Supplementary material for: Pathogenesis of Molar Hypomineralisation: Aged Albumin Demarcates Chalky Regions of Hypomineralised Enamel
Source: Front Physiol. 2020 Sep 30;11:579015. doi: 10.3389/fphys.2020.579015 (PMC7556231; doi:10.3389/fphys.2020.579015)
Supplement: Supplementary file 1 [file Data_Sheet_1.pdf]

V Perez et al.

SUPPLEMENTARY FIGS.

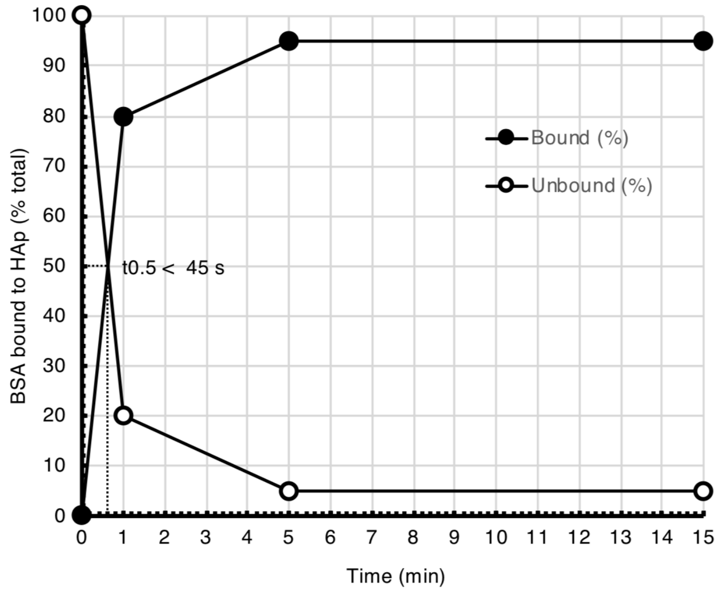

Supplementary Figure S1: Albumin binds hydroxyapatite rapidly and near-quantitatively
Bovine serum albumin (100 µg, from Sigma) was incubated with powdered hydroxyapatite (10 mg, from Sigma; mortar ground) in 100 µl Tris-buffered saline with constant mixing at 20°C for various times as indicated. Following centrifugation (2,000 x g, 2 min), bound- and unbound fractions (volume matched) were analysed with reducing SDS-PAGE and Coomassie staining. Densitometric analysis showed that binding of albumin (BSA) to hydroxyapatite (HAp) was rapid ($t_{0.5}$ < 45 s) and saturable under these conditions.

A

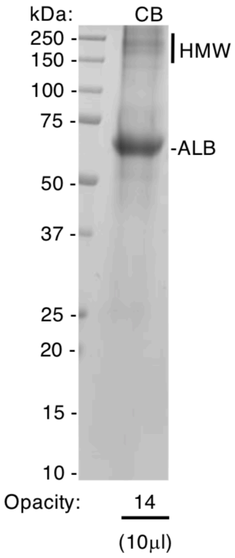

B

Rank	UniProt name	Full name	Mascot score	Peptide hits
1	ALBU_HUMAN	Serum albumin	7914	107
2	F6KPG5_HUMAN	Albumin	7374	102
3	B4DPR2_HUMAN	cDNA FLJ50830 highly similar to serum albumin	6497	85
4	A1AT_HUMAN	Alpha-1-antitrypsin	1264	23
5	K1C10_HUMAN	Keratin type 1 cytoskeletal 10	1031	17

Supplementary Figure S2: Identification of albumin in the HMW band by mass spectrometry
To verify identity of the high-molecular weight, albumin-immunopositive bands from chalky enamel (e.g. HMW, Fig. 3), the ≈ 200–300-kDa Coomassie-stained (CB) gel bands from opacity 14 (HMW) were excised and subjected to peptide mass fingerprinting as described under Methods.

Tandem mass spectrometry data were searched against a human database using the Mascot search engine (Matrix Science, UK) with standard settings.
(A) Coomassie-stained gel showing the HMW bands analysed by mass spectrometry.
(B) The tabulated search results show albumin in the top 3 positions, reflecting much higher Mascot scores and numbers of identified peptides (peptide hits) than the next 2 highest-ranked identifications. These data indicate the HMW bands in **A** are largely comprised of serum albumin.
